# The efficacy and safety of oral and vaginal misoprostol versus dinoprostone on women experiencing labor: A systematic review and updated meta-analysis of 53 randomized controlled trials

**DOI:** 10.1097/MD.0000000000039861

**Published:** 2024-10-04

**Authors:** Mohamed Ramadan, George Bashour, Engy Eldokmery, Amnah Alkhawajah, Karim Alsalhi, Yara Badr, Asmaa Emad, Fatma Labieb

**Affiliations:** a Faculty of Medicine, Suez University, Suez, Egypt; b Faculty of Medicine, Tishreen University, Latakia, Syria; c Cancer Research Center, Tishreen University Hospital, Latakia, Syria; d Faculty of Medicine, Benha University, Qalyubia, Egypt; e College of Medicine, King Faisal University, Al Ahsa, Saudi Arabia; f Faculty of Medicine, Batterjee Medical College, Jeddah, Saudi Arabia; g Faculty of Medicine, Tishreen University, Tartous, Syria; h Faculty of Medicine, Zagazig University, Sharquyah, Egypt; i Faculty of Medicine, Beni-Suef University, Beni-Suef, Egypt.

**Keywords:** dinoprostone, induction of labor, misoprostol, prostaglandin E2, safety

## Abstract

**Background:**

Induction of labor is the process of artificially stimulating the uterus to start labor before the spontaneous onset of labor. It has several medical indications. Commonly used agents are vaginal misoprostol, vaginal prostaglandin E2 (dinoprostone), and oral misoprostol.

**Methods:**

Through October 2023, a literature review was carried out in Cochrane, PubMed, Web of Science, and Scopus to identify randomized clinical studies assessing if oral and vaginal misoprostol has better efficacy of induction of labor over vaginal prostaglandin E2 or dinoprostone as a primary outcome. The data were pooled as mean difference, risk ratio, and 95% confidence interval.

**Results:**

Fifty-three RCTs involving 10,455 patients showed a statistically significant difference in the overall success rate of induction between the misoprostol and prostaglandins E2 (PGE2) groups. They required less additional oxytocin compared to the PGE2 groups. The frequency of tachysystole, uterine hyperstimulation, abnormal cardiotocography, meconium-stained amniotic fluid, and Apgar score <7 at 1 minute were all higher in misoprostol groups than in PGE2 groups. No difference was found in cesarean section, fever, Neonatal Intensive Care Unit admission, or Apgar scores at 1 minute or 5 minutes.

**Conclusion:**

Vaginal misoprostol is more effective at inducing labor but may be less safe than vaginal dinoprostone. Oral misoprostol is generally as safe as vaginal dinoprostone. Vaginal dinoprostone requires lower doses but may need more oxytocin administration.

## 1. Introduction

Induction of labor is the process of artificially stimulating the uterus to start labor before the spontaneous onset of labor by initiation, the process of effacement of the cervix, dilatation of the cervix, and uterine contractions.^[1]^ Recent data demonstrates that over 40% of primiparous women, and over 30% of multiparous women, undergo labor induction.^[2]^

Induction of labor has several medical indications, including gestational age of 41 completed weeks or more, prelabor rupture of amniotic membranes, pregnancy-related hypertension, diabetes, intrauterine growth restriction, premature rupture of membranes after 34 weeks, and other complications^[3]^

Pharmacological agents such as prostaglandins (dinoprostone and misoprostol), are commonly used to reduce the duration of labor and promote vaginal delivery.

Misoprostol, a synthetic analog of prostaglandin E1, has been used widely as a cervical ripening agent for labor induction in obstetrics practice. It binds to uterine smooth muscle cells and increases the strength and frequency of contractions, It stimulates the loss of cervical tone and the breakdown of collagen in the connective tissue of the cervix. Both oral and vaginal misoprostol are useful induction techniques.^[4]^

Prostaglandins E2 (PGE2) preparations have gained wide acceptance for pharmacological induction of labor. Dinoprostone has been used extensively in both Europe and the US, but there are some important safety concerns associated with its use, such as an increased risk of uterine rupture, tachysystole, and hyperstimulation of pregnant women, which may result in fetal hypoxemia and a non-reassuring fetal heart rate (FHR).^[3]^

Vaginal administration of misoprostol compared with vaginal or intracervical administration of dinoprostone gel was associated with a higher rate of vaginal delivery within 24 hours but more uterine hyperstimulation with and without FHR changes^[2]^

The safety and effectiveness of intravaginal misoprostol tablets and dinoprostone vaginal inserts have been compared in numerous clinical trials. Some studies found that misoprostol may be associated with a greater risk of uterine rupture and tachysystole than dinoprostone.^[3]^ The reviews lack a thorough, systematic search across a wide variety of databases. Thus, the objective of this study is to systematically review and meta-analyze to compare misoprostol of any route with dinoprostone in any pregnant woman. Also, to include any updated literature beyond previous meta-analysis studies.

## 2. Materials and methods

This study was structured according to the Cochrane Handbook’s guidelines for systematic reviews and meta-analysis^[5]^ and reporting items for systematic review and meta-analysis (PRISMA-P statement).^[6]^

### 2.1. Literature search strategy

A literature review was carried out in Cochrane, PubMed, Web of Science, Scopus, Embase, and Ovid from inception till June 2024 to identify randomized clinical studies assessing if oral and vaginal misoprostol has better efficacy of induction of labor over vaginal prostaglandin E2 or dinoprostone as a primary outcome and Maternal and fetal safety as a secondary outcome. The search was carried out independently by G.B. and E.M. and the conflict was resolved by consensus among G.B. E.M. and A.A. We used the following terms in our search strategy: (“misoprostol” OR “vaginal misoprostol” OR “misoprostol vaginal insert” OR “vaginal inserts containing misoprostol” OR “vaginal administration of misoprostol” OR “misoprostol through vaginal route” OR “vaginal route of misoprostol” OR “intravaginal misoprostol”) AND (“Dinoprostone” OR “Dinoprostone gel” OR “dinoprostone vaginal gel” OR “dinoprostone vaginal insert” OR “Vaginal dinoprostone” OR “dinoprostone through vaginal route” OR “vaginal administration of dinoprostone” OR “vaginal inserts containing dinoprostone” OR “vaginal route of dinoprostone” OR “intravaginal dinoprostone” OR “intracervical dinoprostone”) OR (“vaginal misoprostol and dinoprostone”).

### 2.2. Study selection

Two reviewers (G. B. and E. M.) checked the full articles to see if they met the criteria. and a third reviewer (M. R.) resolved the conflict. This systematic review included studies that fulfilled the following criteria: Term pregnancy (37–42 weeks), a live Singleton pregnancy, and cephalic presentation. Criteria of excluded studies were signs of fetal distress, previous cesarean section (C-section) (previous uterine surgery), contraindications to vaginal delivery (for example, previa), and fetal anomalies.

### 2.3. Data extraction

The extracted information was the first author’s name, duration of the study, dose for misoprostol, dose for PGE2, route for misoprostol, route for PGE2, and number of patients in each group. Baseline characteristics included age, gestational age, and initial bishop score. Outcome indicators were vaginal delivery at <24 hours, C-section, uterine hyperstimulation, additional oxytocin augmentation, Neonatal Intensive Care Unit (NICU) admission, meconium-stained amino fluid, fever, Apgar score at 1 and 5 minutes, abnormal cardiotocography (CTG), and tachysystole.

### 2.4. Risk of bias and quality assessment

Studies included in the final meta-analysis were assessed by Y.B. and M. R. using the Cochrane collaboration tool for assessing the risk of bias in randomized trials. The quality criteria cover the following points: (1) study design, (2) time frame and sample size, (3) outcome measurement methods, (4) control of variables and to avoid any type of bias; including potential sources such as inadequate randomization generation (selection bias), inadequate allocation (selection bias), lack of blinding of participants (performance bias), lack of blinding of the outcome (detection bias), incomplete outcome data (detection bias), selective reporting (reporting bias), and other sources of bias.

### 2.5. Statistical analysis

Meta-analysis was carried out by A.A. and Y.B. The statistical analysis was performed with RevMan version 5.3 software. Continuous outcomes were pooled as mean difference (MD) and 95% confidence interval (CI) while dichotomous outcomes were pooled as risk ratio (RR) and 95% CIs. The random-effects model was used for analysis and I2 and Chi-square tests were used to detect the heterogeneity (*I*^2^ > 50 indicates significant heterogeneity).

## 3. Results

**Figure 1.**
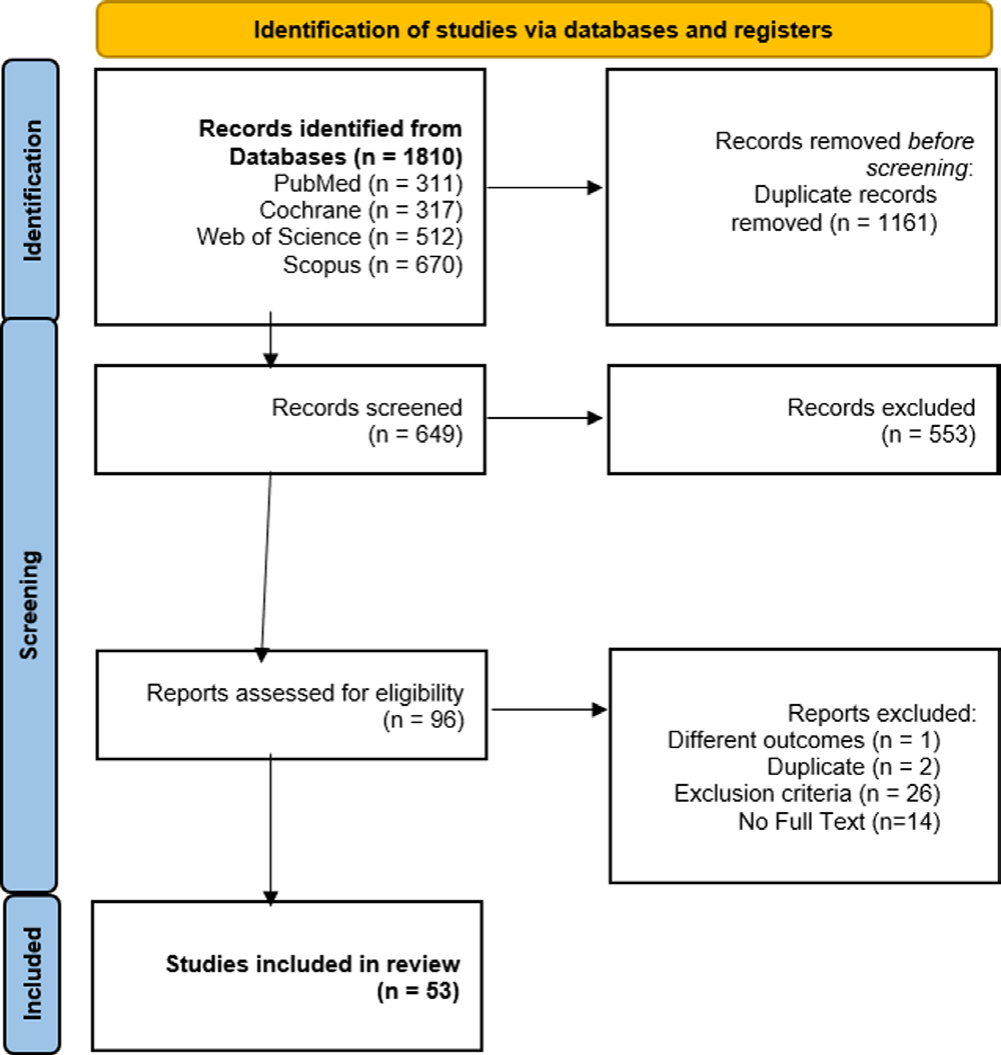
PRISMA flow diagram.

We included 53 RCTs in our meta-analysis including 10,455 patients.^[7–58]^ The included studies spanned a period from 1994 to 2023 and were of different sample sizes ranging from 53 and 650. They were also of different settings taking place in hospitals and birth centers across the globe, including countries like Pakistan, India, United States, Switzerland, United Kingdom, Turkey, Netherlands, Taiwan, Thailand, Canada, Portugal, Australia, France, Jordan, Sudan, Egypt, Greece, Sweden, and Singapore. Information about the selection of the studies can be found in the PRISMA flow diagram (Figure 1).

The majority of the included studies compared misoprostol with PGE2, with both medications administered vaginally. Three studies explored the use of oral misoprostol^[7,8]^ and sublingual misoprostol^[9]^ as an alternative route for labor induction. Summary and baseline characteristics of the included studies are demonstrated in Table 1.

**Table 1:** Summary and baseline characteristics of included studies.

Study ID	Study design	Route of misoprostol	Age		Gestational age		Initial bishop score		Dose of misoprostol	Dose of PGE2	No. of patients	Duration of the study (m)
			Misoprostol	PGE2	Misoprostol	PGE2	Misoprostol	PGE2				
Shaheen 2014	RCT	Oral	25.53 ± 3.974	26.55 ± 3.462	39.23 ± 1.281	39.43 ± 1.201	2.57 ± 1.065	1.98 ± 1.152	25 μg/4 h	2 mg/6 h	106	12
Nagpal 2009^[7]^	RCT	Oral	25.29 ± 2.99	24.63 ± 3.36	38.55 ± 1.15	38.63 ± 1.47	3.81 ± 1.27	3.27 ± 1.12	50 μg/4 h	0.5 mg/6 h	61	13
Dällenbach 2003^[8]^	RCT	Oral	30 ± 5.92	30.33 ± 5.92	40.33 ± 1.69	40.47 ± 1.69	2.77 ± 1.11	3.13 ± 1.04	20 μg/2 h	2 mg/6 h	200	19
Deborah A. Wing 1995^[16]^	RCT	Intravaginal	25.8 ± 6.2	26.2 ±± 6.5	39.7 ± 2.3	40.0 ± 2.4	7.75 ± 3.16	5.75 ± 3.75	25 μg/3 h	0.5 mg/6 h	275	5
John K 1994	RCT	Intravaginal (posterior fornix)	NA	NA	18.75 ± 1.33	17.3 ± 1.6	NA	NA	100 μg/12 h	20 mg	55	NA
Shivarudraiah 2011	RCT	Intravaginal (posterior fornix)	26.5 ± 5.20	27.5 ± 6.36	39.42 ± 1.09	39.43 ± 0.95	3.06 ± 1.59	3.0 ± 1.48	25 μg/6 h	0.5 mg/6 h	320	24
Gemunda 2004	RCT	Intravaginal	31.00 ± 19.26	29.67 ± 19.26	39.00 ± 6.67	39.80 ± 5.11	3.33 ± 5.93	3.00 ± 5.19	25 μg/4 h	1NA 2 mg/6 h	681	36
Perry 1998^[13]^	RCT	Intravaginal	23 ± 6.3	24 ± 5.9	37 ± 2.91	37 ± 2.91	2.2 ± 1.5	1.9 ± 1.6	25 μg/4 h	4 mg/4 h	127	8
Özgür 1996^[12]^	RCT	Intravaginal	27.8 ± 4.3	26.4 ± 5.0	35.5 ± 10	36.5 ± 7	4.0 ± 1.5	3.5 ± 1.5	100 μg	0.5 mg	65	NA
Hösli 2007^[58]^	RCT	Intravaginal	30.3 ± 6.2	31.5 ± 5.8	40.9 ± 1.2	40.8 ± 1.4	2.7 ± 1.4	2.4 ± 1.7	100 µg/24 h	2 mg/12 h	107	18
Samanta 2023^[11]^	RCT	Intravaginal	26.69 ± 2.51	26.74 ± 2.99	26.74 ± 2.99	8.36 ± 1.13	NA	NA	100 µg/24 h	0.5 mg	140	24
Garg 2021^[10]^	RCT	Intravaginal	26.63 ± 3.601	26.24 ± 4.003	37.9 ± 0.99	38.1 ± 1.15	2.41 ± 1.09	2.92 ± 1.1	25 μg	0.5 mg	150	NA
Wing1997^[18]^	RCT	Intravaginal (post fornix)	NA	NA	39.5 ± 2.4	39.2 ± 2.3	NA	NA	25 μg	10 mg	197	9
Subrek 1997	RCT	Intravaginal	28.8 ± 5.4	30.4 ± 4.7	40 ± 1.63	40 ± 2.0	2.4 ± 1.35	3.0 ± 1.64	50 μg	3 mg	100	11
Sanchez NA Ramos 1998^[19]^	RCT	Intravaginal	24.4 ± 6.8	23.1 ± 5.9	38.8 ± 2.7	38.7 ± 2.6	NA	NA	50 μg	10 mg	223	9
Rowlands 2001^[21]^	RCT	Intravaginal	28.9 ± 5.4	27.9 ± 4.4	40.95 ± 1	40.72 ± 1.18	3.3 ± 1.3	3.5 ± 1.5	100 μg	2 mg	125	20
Rozenberga 2001^[20]^	RCT	Intravaginal	29.3 ± 4.67	29.6 ± 4.59	37.13 ± 4.22	39.7 ± 2.07	NA	NA	50 μg	2 mg	369	21
Ramsey 2003^[22]^	RCT	Intravaginal	27.9 ± 4.6	28.0 ± 4.4	39.3 ± 1.6	39.2 ± 1.3	3.0 ± 1.1	3.0 ± 1.2	50 μg	0.5 mg gel 10 mg vaginal insert	111	16
Pandis 2001^[14]^	RCT	Intravaginal	29.5 ± 5.5	29.7 ± 5.4	40.6 ± 1.2	40.6 ± 1.2	3 ± 1	3.1 ± 1	50 μg	2 mg	435	12
Nunes 1999^[23]^	RCT	Intravaginal	27.1 ± 5.1	27.3 ± 5.1	40.0 ± 1.3	40.1 ± 1.2	3 ± 2.96	2.67 ± 3.70	100 μg	2 mg	189	NA
Mundule 1996	RCT	Intravaginal	27.6 ± 5.1	27.4 ± 5.5	40.91 ± 1.11	40.78 ± 1.25	3.67 ± 2.22	4 ± 2.96	50 μg	0.5 mg intracervical 1 or 2 mg intravaginal	222	7
Lokugamage 2003^[33]^	RCT	Intravaginal	29.33 ± 19.26	30 ± 21.48	39.33 ± 4.44	39.67 ± 5.19	3.67 ± 5.19	4 ± 4.44	50 μg	2 mg	191	12
Kumar 2001^[25]^	RCT	Intravaginal	22 ± 5.8	22 ± 5.5	39 ± 2.8	38.2 ± 2.5	NA	NA	25 μg	0.5 mg	200	12
Kandali 1996	RCT	Intravaginal	22.3 ± 5.7	22.5 ± 5.3	38.2 ± 3.4	38.8 ± 2.8	4 ± 1.4	3.8 ± 1.4	100 μg	NA	224	6
Herbutya 1997	RCT	Posterior fornix	29.12 ± 4.69	28.18 ± 4.72	39.33 ± 1.41	39.74 ± 1.43	2.22 ± 1.06	2.5 ± 1.15	100 μg	1.5 mg	110	11
Gary 2009	RCT	Intravaginal	30.7 ± 5.8	30.6 ± 5.7	39.2 ± 2.5	39.2 ± 4.2	NA	NA	50 μg	10 mg	186	15
Danielian 1999^[28]^	RCT	Intravaginal	28.67 ± 19.26	27.33 ± 17.04	40.33 ± 2.96	40 ± 3.70	4 ± 4.44	4.33 ± 3.70	50 μg	1 mg	211	NA
Chang 2003^[29]^	RCT	Intracervical	30.7 ± 4.9	29.6 ± 3.9	39.5 ± 1.7	39.9 ± 1.1	2.8 ± 1.0	2.6 ± 1.1	50 μg	0.5 mg	86	13
Buser 1997^[31]^	RCT	Intravaginal	27.7 ± 5.6	27.1 ± 5.8	39.2 ± 1.9	39.3 ± 1.8	2.66 ± 1.3	2.64 ± 1.4	50 μg	0.5 mg	155	17
Blanchette 1999^[30]^	Comparison prospective	Posterior fornix	29.8 ± 5.5	29.5 ± 5.1	40.2 ± 1.4	40.4 ± 1.2	3.1 ± 1.3	2.9 ± 1.2	25 to 50 μg	0.5 mg	226	Two studies compared together (12 months, 12 months)
Agarwal 2003^[32]^	Prospective clinical trials	Intravaginal	26.5 ± 4.0	26 ± 3.7	38.5 ± 1.2	37.8 ± 1.2	3.8 ± 1.3	3.8 ± 1.1	50 μg	0.5 mg	120	8
David C Young 2020^[48]^	RCT	Intravaginal	28.8 ± 5.6	29.1 ± 5.7	39.4 ± 1.4	40.0 ± 1.5	4.1 ± 1.9	4.2 ± 2.1	50 μg	1 mg	344	9
Madaan 2014^[56]^	RCT	Intravaginal	26.5 ± 5.3	25.5 ± 4.7	39.54 ± 1.26	39.48 ± 1.50	3.86 ± 1.51	3.9 ± 1.38	100 μg	0.5 mg	100	7
Chaudhuri 2011^[55]^	RCT	Intravaginal	23.02 ± 3.59	23.09 ± 3.54	38.40 ± 0.85	38.26 ± 0.87	NA	NA	25 μg	0.5 mg	207	25
Gulshan A. Saeed 2009	RCT	Posterior fornix	26.22 ± 3.40	26.22 3.40	40.11 ± 1.37	40.11 ± 1.37	3.12 ± 1.28	3.12 ± 1.28	50 μg	3 mg	208	NA
Rajiv Mahendru 2011^[54]^	RCT	Intravaginal	22.5 ± 2	22.9 ± 2	38.5 ± 1	38.6 ± 1	3.3 ± 2.4	3.3 ± 2.4	25 μg	0.5 mg	111	8
TNAC Tan 2010^[51]^	RCT	Intravaginal	29.95 ± 4.43	31.42 ± 5.19	39.65 ± 1.26	39.38 ± 1.35	2.67 ± 1.06	2.7 ± 0.96	25 μg	0.5 mg	115	23
Özkan 2009^[42]^	RCT	Intravaginal	NA	NA	NA	NA	NA	NA	50 μg	10 mg	112	18
AA Calder 2008^[49]^	Comparative study	Intravaginal	30.55 ± 4.36	30.85 ± 4.47	40.6 ± 1.6	40.6 ± 1.5	4.5 ± 1.62	4.25 ± 1.13	25 μg	3 mg	626	27
M Prager 2008^[37]^	RCT	Vaginally posterior fornix	32.2	33.3	40.2	40.3	NA	NA	25 μg	2 mg	390	24
Smiti Nanda 2007^[43]^	Comparative study	Intravaginal	22.72 ± 2.55	23.067 ± 3.11	39.98 ± 1.34	39.357 ± 1.47	2.74 ± 1.24	2.66 ± 1.41	25 μg	0.5 mg	100	NA
S. Sifakis 2006^[35]^	RCT	Intravaginal	38.95 ± 0.83	27.5 ± 3.18	38.95 ± 0.84	39.3 ± 0.84	2.15 ± 0.32	2.3 ± 0.32	50 μg	3 mg	415	37
Preutthipan 2006^[36]^	RCT	Posterior fornix	33.2 ± 4.6	32.2 ± 4.7	NA	NA	NA	NA	200 μg	3 mg	310	48
Meyer 2005^[44]^	RCT	Intravaginal	30.3 ± 0.1	29.9 ± 1.3	39.5 ± 0.2	39.8 ± 0.2	3 ± 0.41	3.5 ± 0.41	0.25 μg	0.5 mg	83	25
Patrick S. Ramsey2005^[40]^	RCT	Intravaginal	27.5 ± 8.6	27.1 ± 7.6	20.4 ± 2.1	20.0 ± 2.6	NA	NA	50 μg	0.5 mg	126	28
AYYAD 2002	RCT	Intravaginal	NA	NA	NA	NA	NA	NA	50 μg	0.5 mg mixed/methylcellulose	238	12
Papanikolaou 2004^[41]^	RCT	Posterior fornix	NA	NA	NA	NA	NA	NA	50 μg	3 mg	163	27
Rozenberg 2003^[39]^	RCT	Posterior fornix	29.25 ± 1.17	28.75 ± 0.84	40.8 ± 0.7	40.7 ± 0.9	3 ± 0.41	3 ± 0.41	50 μg	10 mg	200	12
Megalo 2004^[45]^	RCT	Intravaginal	29 ± 4	30 ± 5	40.6 ± 1.5	40.4 ± 1.5	4.2 ± 1.7	4.4 ± 1.7	50 μg	0.5 mg	200	18
S. Ramsey 2004^[38]^	RCT	Intravaginal	28.0 ± 4.6	28.0 ± 4.4	39.3 ± 1.6	39.7 ± 1.2	3 ± 0.82	3 ± 1.03	600 μg	10 mg with oxytocin infused in 500 mL NS	120	37
M. Elhassan 2003^[53]^	RCT	Intravaginal	26.4 ± 3.6	27.7 ± 4.3	40.8 ± 1.7	41.0 ± 1.3	NA	NA	50 μg	3 mg	120	9
Makhlouf 2003^[46]^	RCT	Intravaginal	27.8 ± 1	25.4 ± 1	23.3 ± 0.7	23.4 ± 0.8	6 ± 8.1	11.2 ± 14.8	100 μg	6 mg	80	12
Vimarshitha P 2023^[52]^	RCT	Intravaginal	NA	NA	NA	NA	2.65 ± 0.78	2.25 ± 0.60	NA	NA	180	NA

### 3.1. Risk of bias and quality assessment

**Figure 2.**
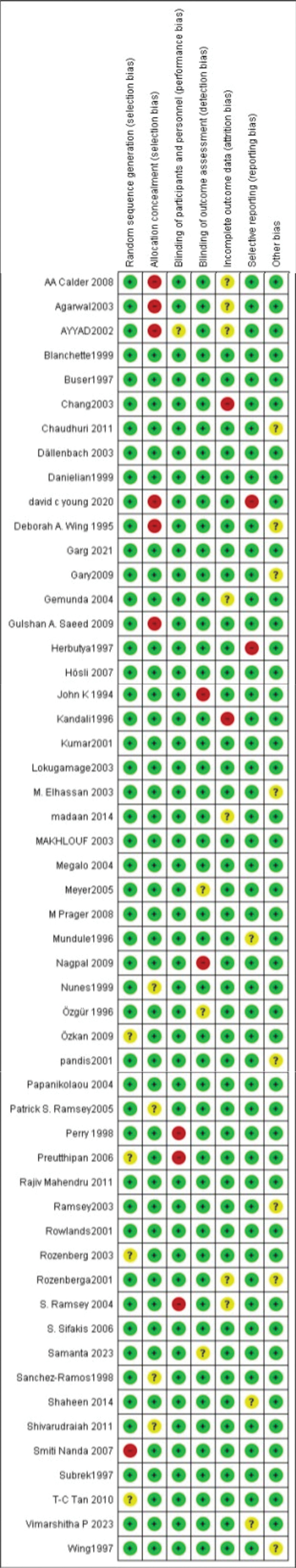
Risk of Bias for each included study Figure.

**Figure 3.**
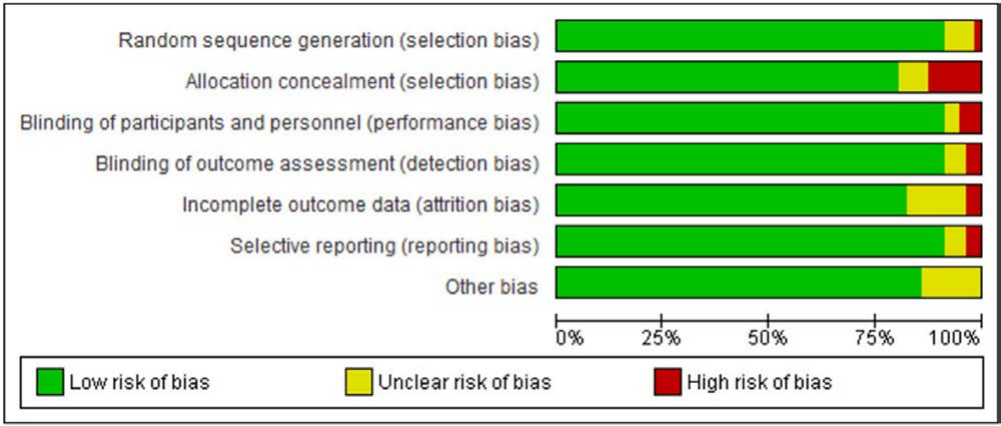
Risk of Bias summary.

The Cochrane risk of bias tool (Rob2) was used to evaluate the quality of each included study (Figs. 2 and 3). All included studies had a low risk of bias related to random sequence generation, except for one study which had a high risk.^[43]^ Four studies indicated unclear risk of bias.^[20,36,42,51]^ The majority of included studies had a low risk of bias related to allocation concealment bias, with the exception of 6 studies which had a high risk.^[16,32,47–50]^ Four studies indicated unclear risk of bias.^[19,23,40,57]^ While most of the included studies showed a low risk of bias related to blinding of participants and personnel bias, there was the exception of 3 studies which were evident with a high risk,^[13,36,38]^ also one study^[50]^ which indicated unclear risk of bias. As for blinding of outcome assessment, bias all studies presented with low risk, with the exception of 2 studies^[7,17]^ that showed a high risk of bias, and 3 studies^[11,12,44]^ that came with unclear risk of bias. Low risk of incomplete outcome data was predominant among the included studies with the exception of 2 studies that had high risk,^[26,29]^ and 7 studies that had unclear risk of bias.^[15,20,32,38,49,50,56]^ When it came to selective reporting bias, the bulk of the included studies showed low risk, however, 2 studies had high risk,^[34,48]^ and 3 studies^[9,24,52]^ had unclear risk of bias. Other biases including funding and conflict of interests were represented by low risk in the majority of included studies, with only 7 studies having unclear risk of bias.^[14,18,20,22,27,53,55]^ Overall, most studies had a fairly low risk of bias except for one study which had a high overall risk of bias.^[50]^

## 4. Outcomes

### 4.1. Efficacy outcomes

#### 4.1.1. Induction success

**Figure 4.**
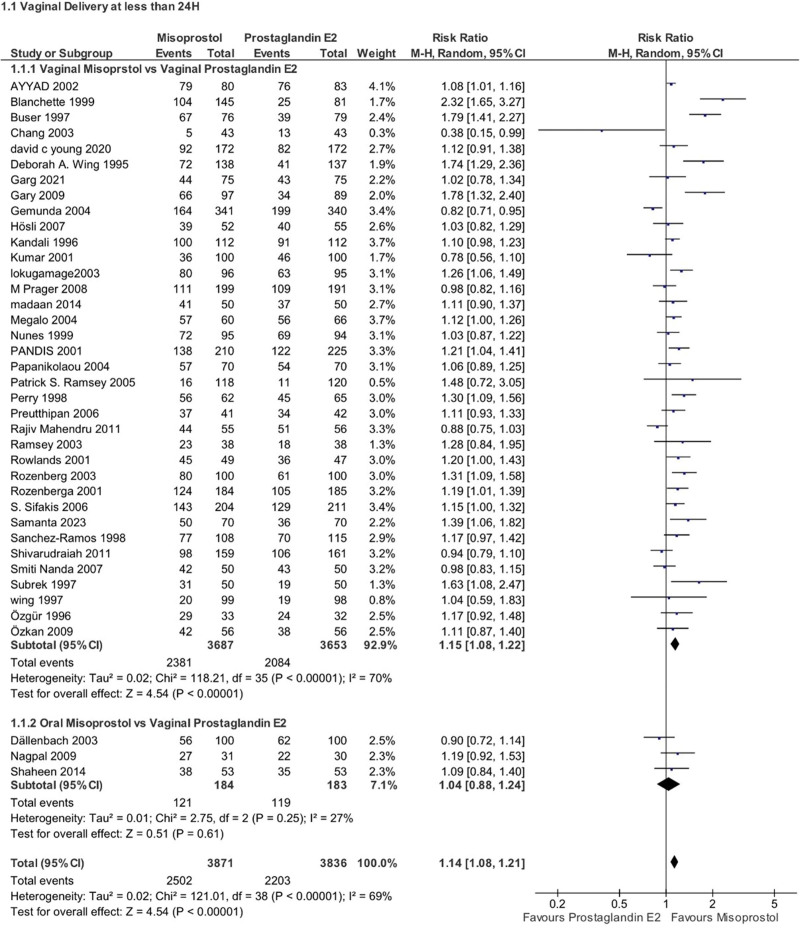
Forest plot presenting OR for the effect of misoprostol versus dinoprostone on vaginal delivery rate in <24 hours.

Our meta-analysis revealed a statistically significant difference in the overall success rate of induction between the misoprostol and PGE2 groups (RR 1.14, 95% CI 1.08–1.21, *P* < .00001, *I*^2^ = 69%), favoring misoprostol. Subgroup analysis based on the route of misoprostol administration yield a significant difference when comparing vaginal misoprostol to vaginal PGE2 (RR 1.15, 95% CI 1.08–1.22, *P* < .00001, *I*^2^ = 70%), while no significant difference was observed between oral misoprostol and vaginal PGE2 (RR 1.04, 95% CI 0.88–1.24, *P* = .61, *I*^2^ = 27%) (Fig. 4).

### 4.2. Safety outcomes

#### 4.2.1. Need for additional oxytocin

**Figure 5.**
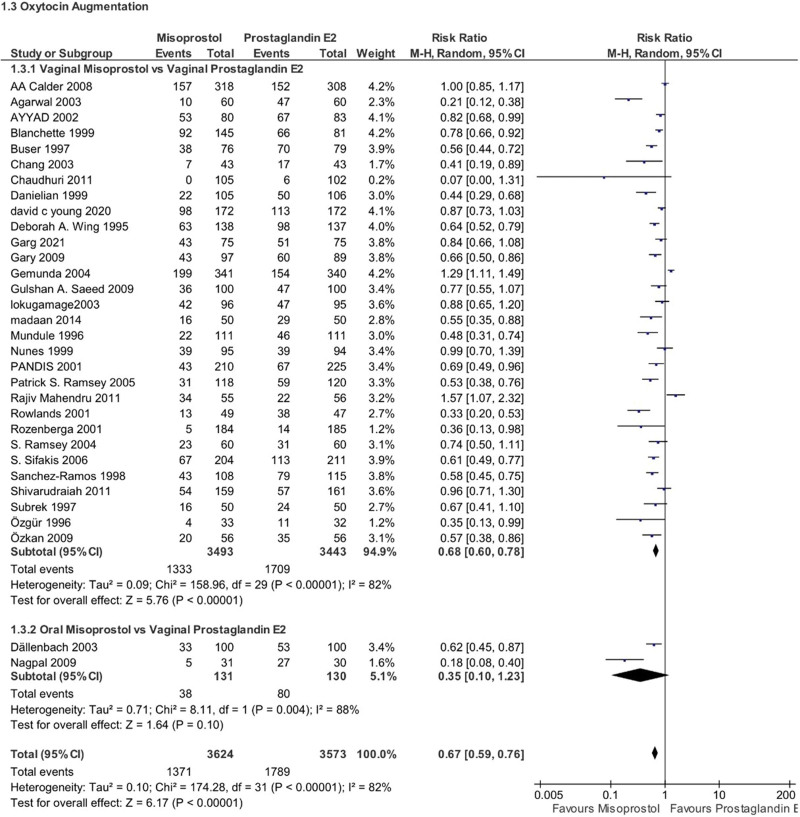
Forest plot presenting OR for the effect of misoprostol versus dinoprostone on the need of additional oxytocin.

Patients who administered misoprostol appeared to be requiring less additional oxytocin compared to PGE2. Changes were significant (RR 0.67, 95% CI 0.59–0.76, *P* < .00001, *I*^2^ = 82%). subgroup analysis showed that when compared to vaginal PGE2, patients who needed additional oxytocin were significantly fewer than those with vaginal misoprostol (RR 0.68, 95% CI 0.60–0.78, *P* < .00001, *I*^2^ = 82%), whereas changes were nonsignificant for the oral group. (RR 0.35, 95% CI 0.10–1.23, *P* = .10, I2 = 88%) (Fig. 5).

#### 4.2.2. Tachysystole

**Figure 6.**
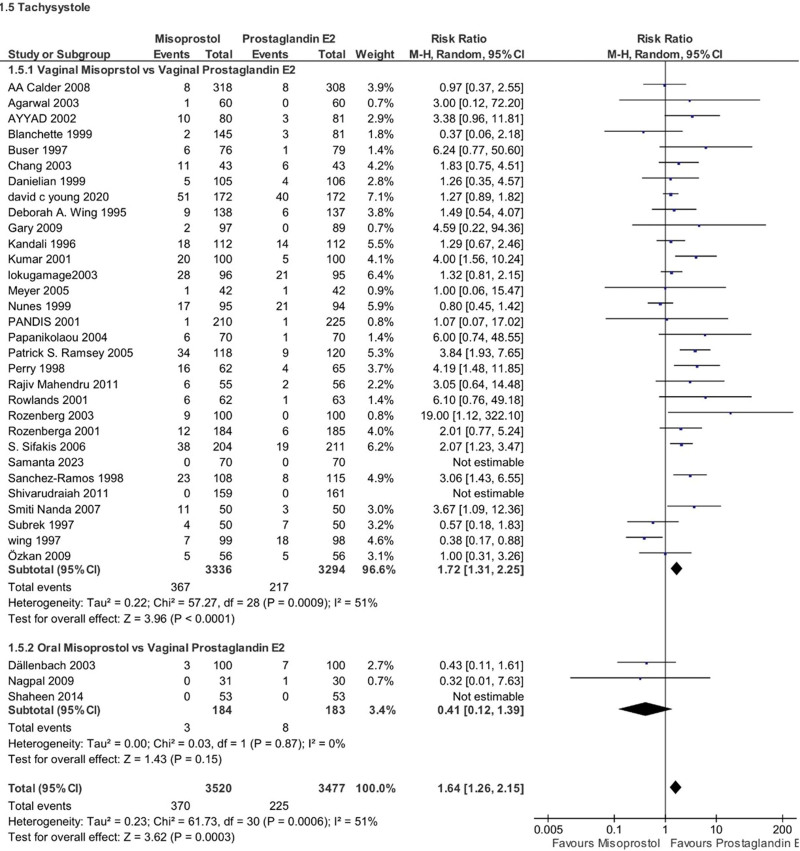
Forest plot presenting OR for the effect of misoprostol versus dinoprostone on tachysystole.

PGE2 was associated with a lower risk of mothers developing tachysystole during labor compared to misoprostol (RR 1.64, 95% CI 1.26–2.15, *P* = .0003, I2 = 51%). Subgroup analysis showed that the risk was higher for women who received vaginal misoprostol compared to vaginal PGE2 (RR 1.72, 95% CI 1.31–2.25, *P* < .00001, *I*^2^ = 82%). No significant difference was observed for women who received oral misoprostol (RR 0.41, 95% CI 0.12–1.39, *P* = .15, *I*^2^ = 0%) (Fig. 6).

### 4.3. Uterine hyperstimulation

**Figure 7.**
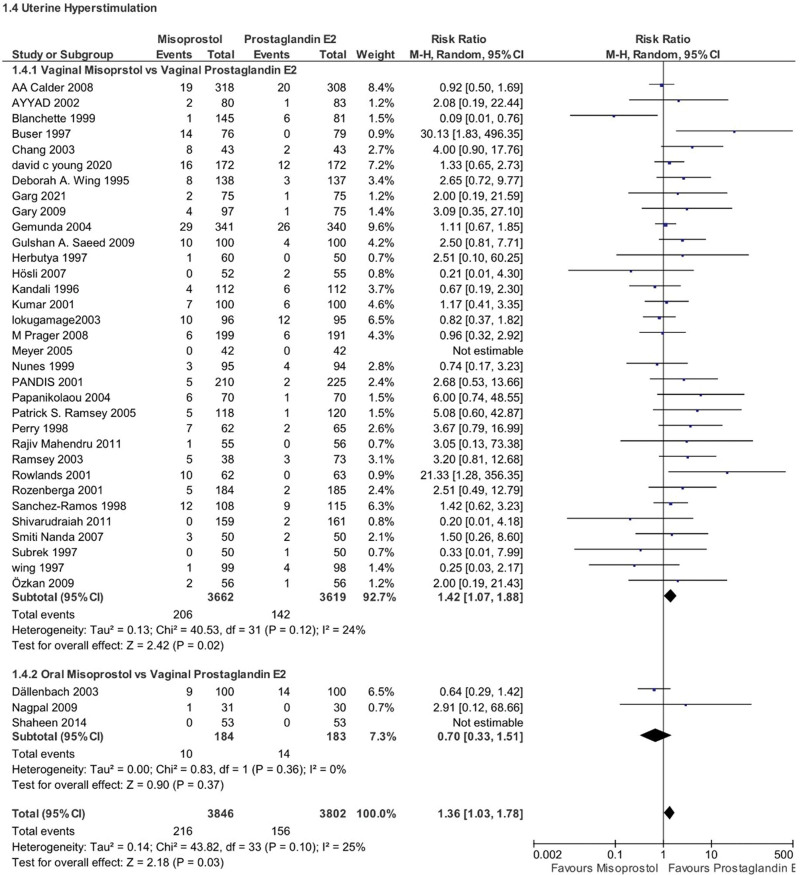
Forest plot presenting OR for the effect of misoprostol versus dinoprostone on uterine hyperstimulation.

Misoprostol use was associated with a significantly higher risk of uterine hyperstimulation compared to PGE2 (RR 1.36, 95% CI 1.03–1.78, *P*-value = .03, *I*^2^ = 25%). Once again, this risk was more for women who received vaginal misoprostol compared to vaginal PGE2 (RR 1.42, 95% CI 1.07–1.88, *P* = .02, *I*^2^ = 24%), with no significant difference observed for the oral route (RR 0.70, 95% CI 0.33–1.51, *P* = .37, *I*^2^ = 0%) (Fig. 7).

### 4.4. C-section rates

**Figure 8.**
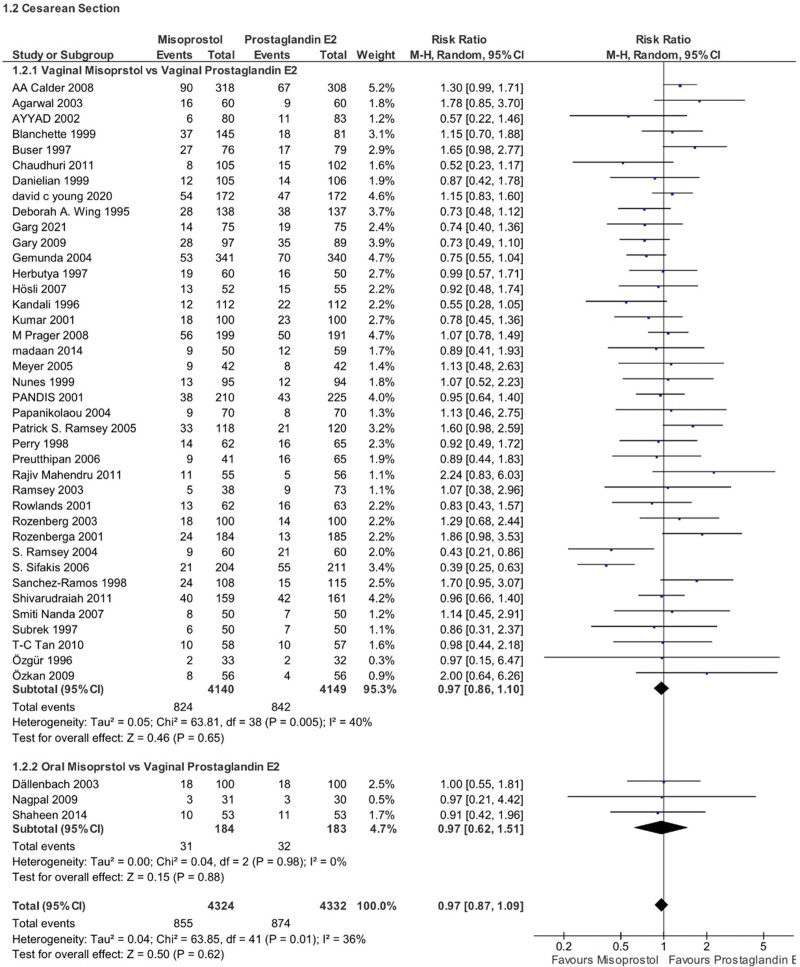
Forest plot presenting OR for the effect of misoprostol versus dinoprostone on cesarean section rate.

There was no significant difference in the overall C-section rates between PGE2 and misoprostol groups (RR 0.97, 95% CI 0.87–1.09, *P* = .62, *I*^2^ = 36%). Subgroup analysis by misoprostol route of administration was also nonsignificant for both the vaginal group (RR 0.97, 95% CI 0.86–1.1, *P* = .65, *I*^2^ = 40%) and the oral group. (RR 0.97, 95% CI 0.62–1.51, *P* = .88, *I*^2^ = 0%) (Fig. 8).

### 4.5. Newborn outcomes

#### 4.5.1. NICU admission

**Figure 9.**
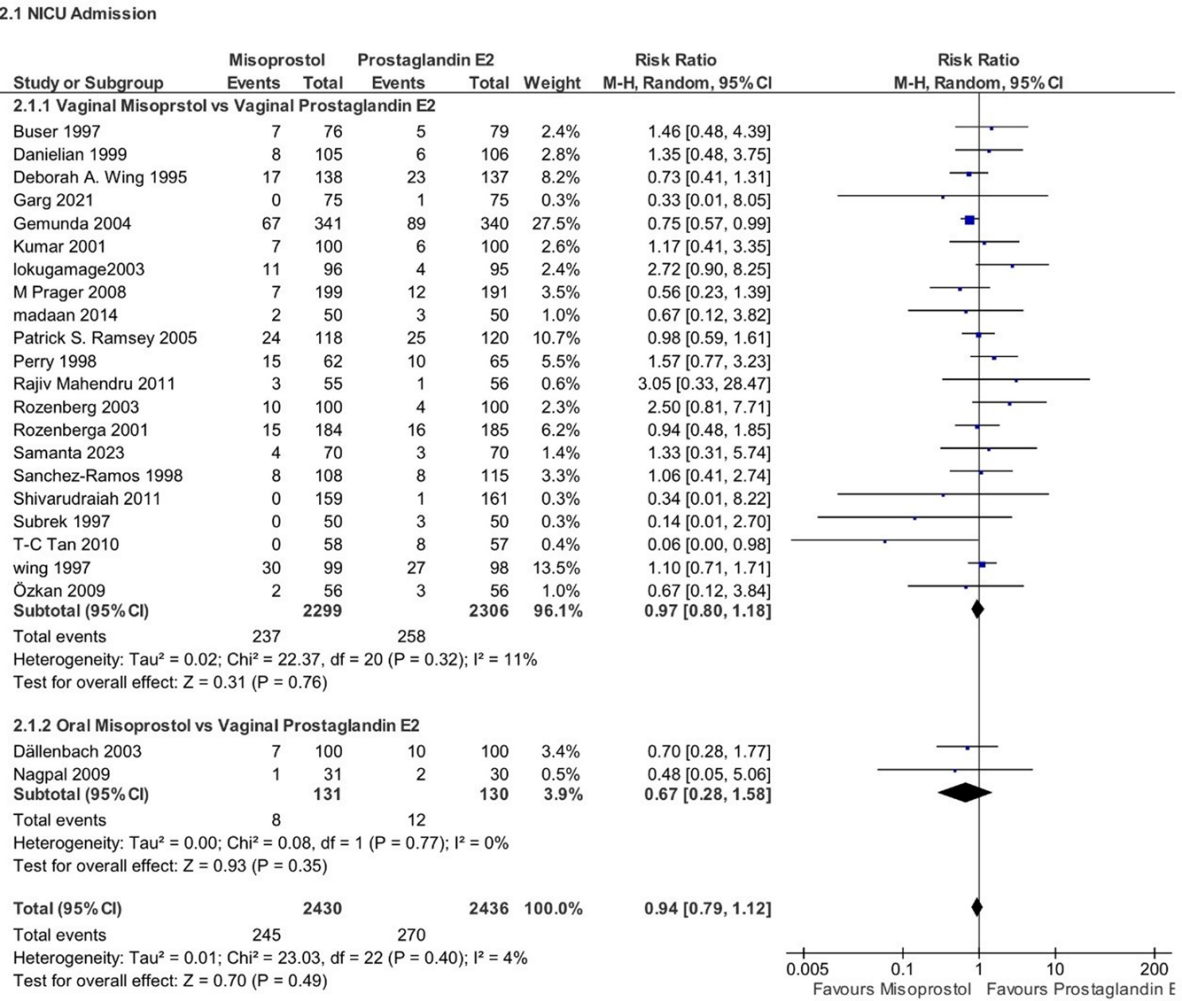
Forest plot presenting OR for the effect of misoprostol versus dinoprostone on NICU Admission. NICU = Neonatal Intensive Care Unit.

The overall rate of needing intensive care after birth (NICU admission) for newborns was not statistically different between misoprostol and PGE2 groups (RR 0.94, 95% CI 0.79–1.12, *P* = .49, *I*^2^ = 4%). subgroup analysis by misoprostol route of administration was nonsignificant for both the vaginal group (RR 0.97, 95% CI 0.80–1.18, *P* = .76, *I*^2^ = 11%) and oral group (RR 0.67, 95% CI 0.28–1.58, *P* = .35, *I*^2^ = 0%) (Fig. 9).

#### 4.5.2. Apgar scores

**Figure 10.**
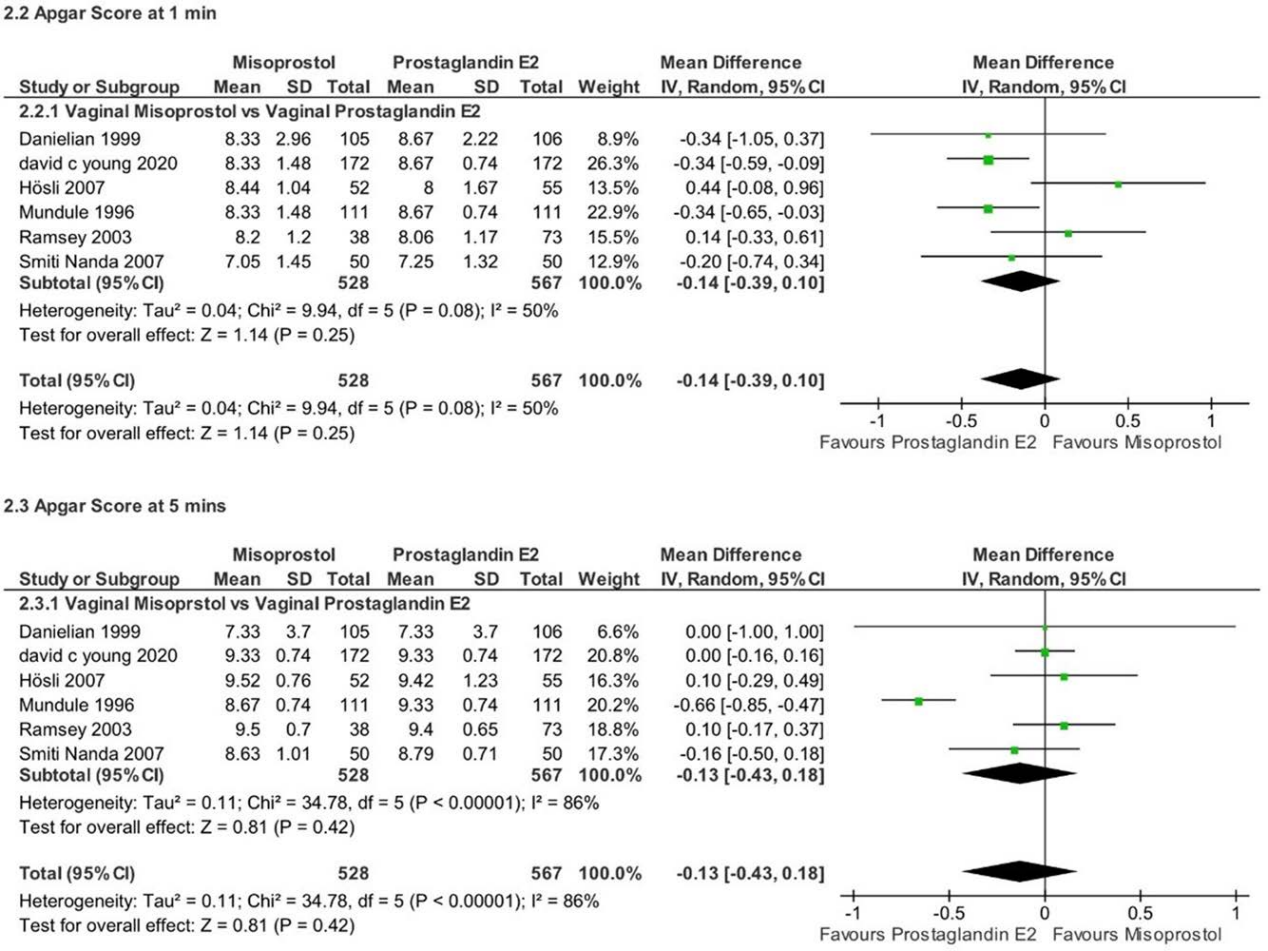
Forest plot presenting MD for the effect of misoprostol versus dinoprostone on Apgar score at 1 minute and 5 minutes. MD = mean difference.

There were no significant differences between the 2 groups in average Apgar scores at 1 or 5 minutes after birth (MD ‐0.14, 95% CI ‐0.39 to 0.10, *P* = .25, *I*^2^ = 50% for 1 minute; MD ‐0.13, 95% CI ‐0.43 to 0.18, *P* = .42, *I*^2^ = 86% for 5 minutes). Subgroup analysis by misoprostol route (vaginal vs oral) was not applicable for these outcomes (Fig. 10).

**Figure 11.**
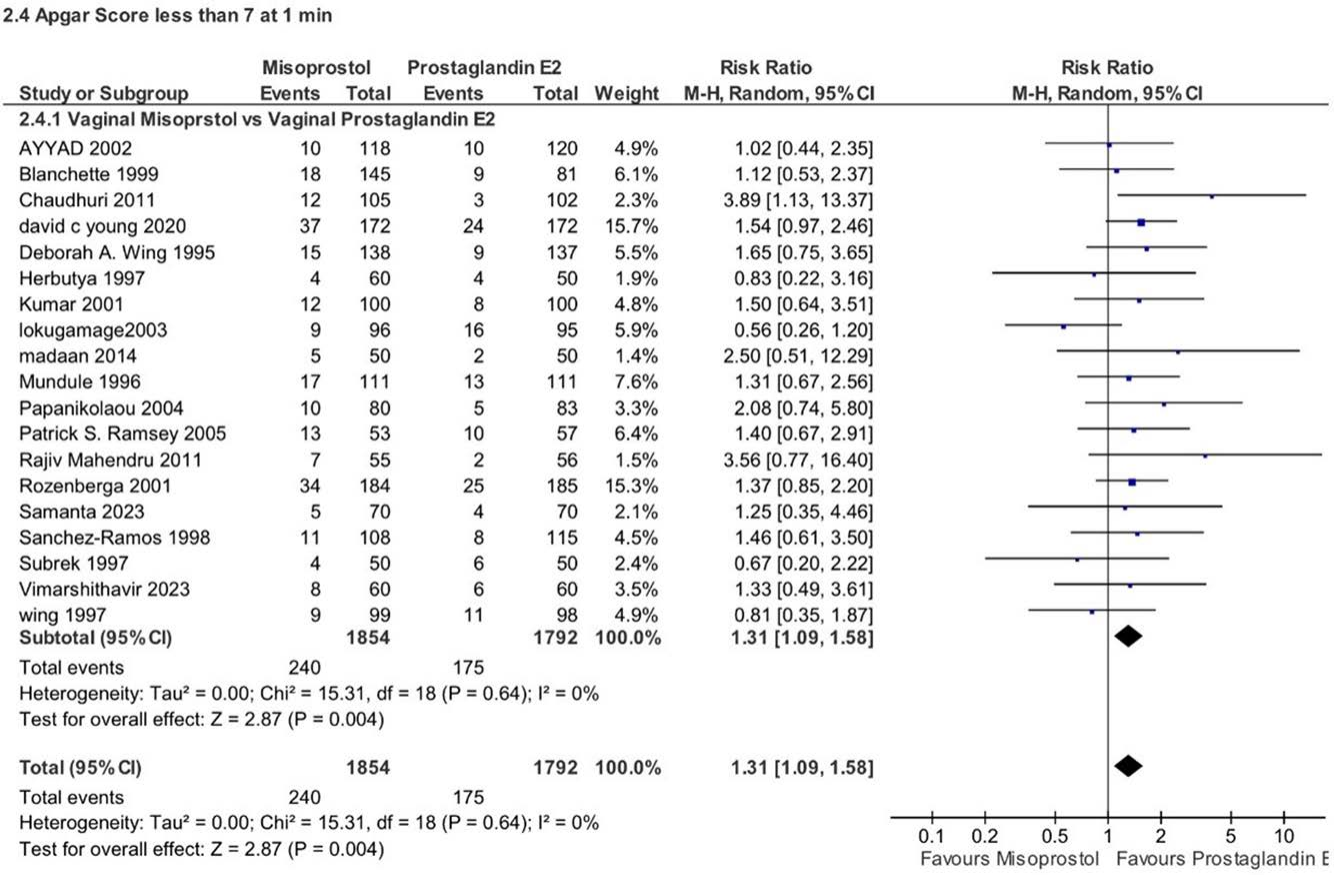
Forest plot presenting OR for the effect of misoprostol versus dinoprostone on outcome “Apgar store less than 7 at 1 min”.

**Figure 12.**
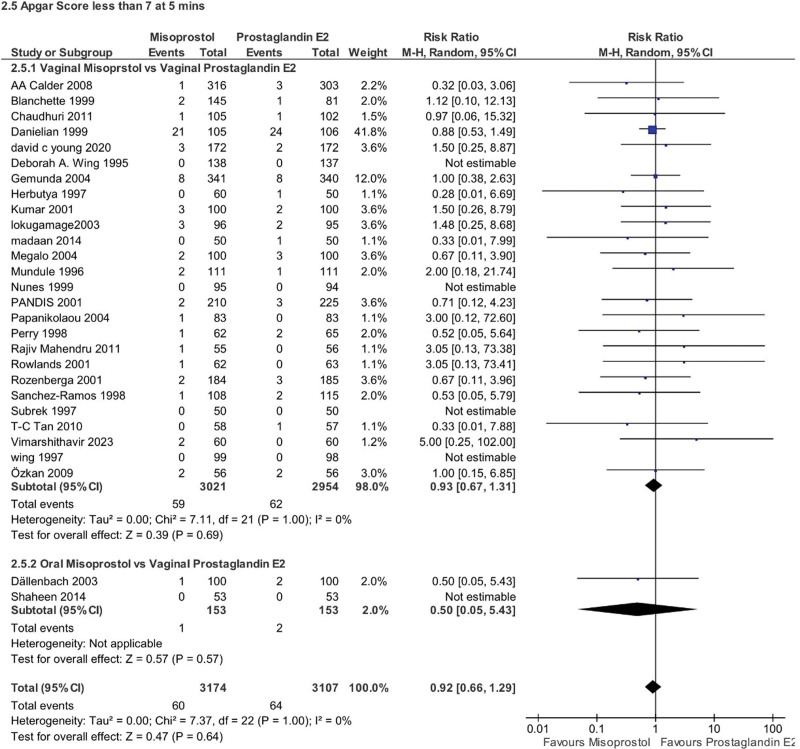
Forest plot presenting OR for the effect of misoprostol versus dinoprostone on outcome “Apgar store less than 7 at 5 mins”.

Newborns to mothers who received misoprostol had a slightly higher risk of having a low Apgar score (<7) at 1 minute compared to those born to mothers who received PGE2 (RR 1.31, 95% CI 1.09–1.58, *P* = .004, *I*^2^ = 64%). However, there was no such difference at the 5-minute mark (RR 0.92, 95% CI 0.66–1.29, *P* = .64, *I*^2^ = 0%). Subgroup analysis was not applicable for these specific Apgar scores either (Figs. 11 and 12).

#### 4.5.3. Other newborn findings

**Figure 13.**
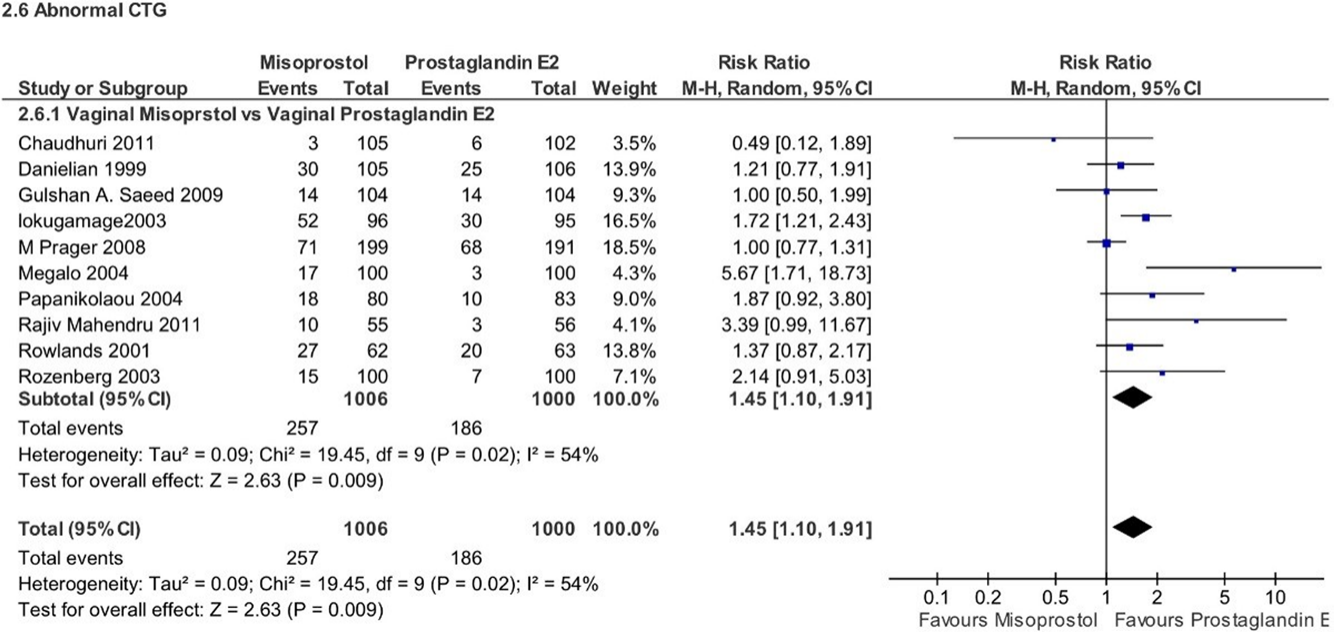
Forest plot presenting OR for the effect of misoprostol versus dinoprostone on abnormal CTG. CTG = cardiotocography.

**Figure 14.**
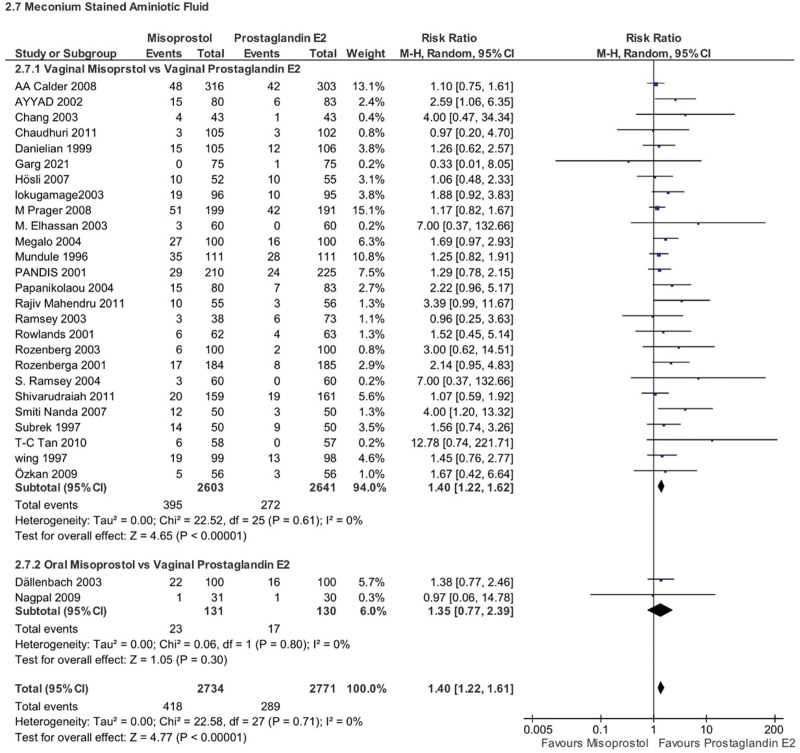
Forest plot presenting OR for the effect of misoprostol versus dinoprostone on meconium stained AF.

Misoprostol use was associated with a higher risk of 2 other potential newborn complications: (1) abnormal CTG readings (RR 1.45, 95% CI 1.10–1.91, *P* = .009), (2) meconium-stained amniotic fluid (RR 1.40, 95% CI 1.22–1.61, *P* < .00001, *I*^2^ = 0%). However, for meconium-stained amniotic fluid, the subgroup analysis revealed that vaginal misoprostol showed a clear association with this complication compared to vaginal PGE2 (RR 1.40, 95% CI 1.22–1.62, *P* < .00001, *I*^2^ = 0%), there was no significant difference between oral misoprostol and vaginal PGE2 (RR 1.35, 95% CI 0.77–2.39, *P* = .30, *I*^2^ = 0%) (Figs. 13 and 14).

### 4.6. Side effects

#### 4.6.1. Fever

**Figure 15.**
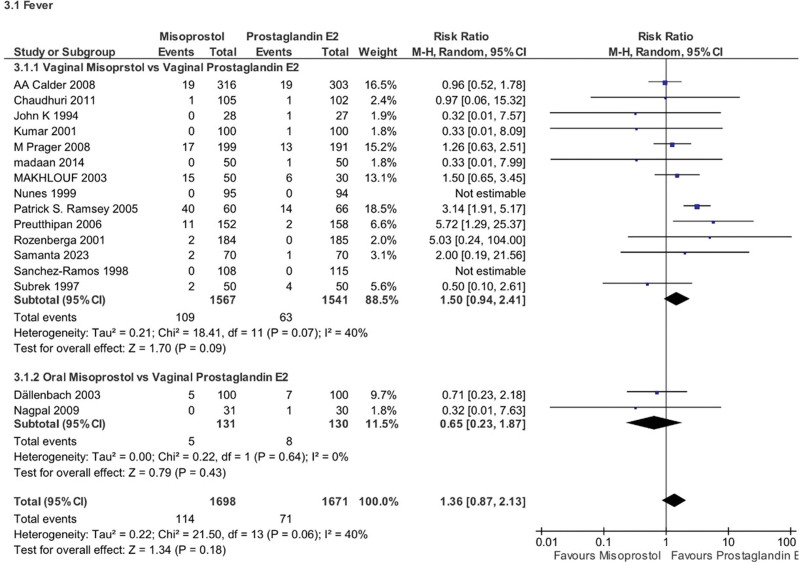
Forest plot presenting OR for the effect of misoprostol versus dinoprostone on fever.

No significant difference was found in the risk of fever between the 2 groups (RR 1.36, 95% CI 0.87–2.13, *P* = .18, *I*^2^ = 40%). Subgroup analysis by misoprostol route showed similar results for both subgroups (Fig. 15).

## 5. Discussion

This meta-analysis included more studies than any previous meta-analyses as we included more RCTs in our analysis totaling 53 studies than the study conducted by Taliento et al Which only included 39 RCTs.^[3]^ Also, we are the first study to compare both oral and vaginal misoprostol to prostaglandin E2 or dinoprostone, furthermore, the selection of trials was according to specific inclusion criteria mentioned in the methods section. Finally, our analysis included new outcomes in terms of fetal safety like Apgar scores at 1 and 5 minutes, meconium-stained amniotic fluid, and fever.

The fact that we analyzed studies with the different routes of administration of misoprostol has enabled us to find new outcomes. Unlike studies included in some previous meta-analyses like Taliento et al, most of our included studies were of low overall risk of bias.^[3]^

Although Vaginal Misoprostol appears to be a more powerful labor inducer than dinoprostone, it seems to be less safe. Vaginal Misoprostol significantly resulted in more frequency of vaginal delivery in <24 hours and lower frequency of the need for additional oxytocin. However, it also showed significantly higher frequencies of outcomes like tachysystole, uterine hyperstimulation, abnormal CTG, and meconium-stained amniotic fluid. And there was no significant difference when it came to the rate of cesarian delivery. Also, most neonatal outcomes like NICU admission, Apgar score at 1 minute, Apgar score at 5 minutes, and Apgar score <7 at 5 minutes were of no significant difference across the 2 groups.

Interestingly, when compared to vaginal dinoprostone, oral misoprostol showed no significant difference in most of the outcomes like vaginal delivery in <24 hours, and the need for additional oxytocin. It also showed no difference regarding the frequency of adverse effects such as tachysystole and uterine hyperstimulation. This suggests that using oral misoprostol can be similar to the impact of vaginal dinoprostone without the risk of invasiveness and also less risk of peripartum infections.

Our findings of vaginal misoprostol being a more powerful agent are consistent with previous meta-analyses.^[2]^ They also found it reduced the need for additional oxytocin Liu et al also found no significant difference when it came to the rate of cesarian delivery across the 2 vaginal groups.

Most of our included studies used misoprostol of 50 mcq or higher, yet misoprostol was associated with a significantly higher frequency of abnormal CTG. The American Congress of Obstetricians and Gynecologists declares that the use of higher doses of misoprostol (50 mcq every 6 hours) was associated with a higher rate of FHR decelerations.^[59]^

Although there was no significant difference in the rate of NICU admission when comparing vaginal misoprostol and dinoprostone, these outcomes along with other complications appear to be of lower rates when using lower doses of misoprostol. A previous meta-analysis by McMaster et al showed that lower doses of misoprostol (25 mcq) were associated with lower rates of NICU admission when compared to higher doses (50 mcq).^[60]^

The IMPROVE trial compared oral vs vaginal misoprostol for labor induction, they found a significantly lower rate of FHR abnormalities and cesarean deliveries in the vaginal group than oral group, they used 100 mcq divided into 2 or 4 doses, while doses of oral misoprostol in the 3 studies included in our analysis ranged from 20 to 50 mcq.^[59]^

The pharmacokinetics of oral oral labor inducers are different from vaginal agents. Oral misoprostol is rapidly cleared from plasma and is of shorter half-life when compared to vaginal agents^[61]^ and therefore, oral agents tend to cause fewer side effects. Since Oral misoprostol shows no statistical difference in labor induction when compared to vaginal dinoprostone it may be safer to use oral misoprostol and avoid potential side effects that can be caused by vaginal agents in some cases if indicated. these findings align well with previous studies^[62]^ Monika et al also suggested that the use of oral misoprostol is generally more effective than using vaginal PGE2 or dinoprostone.^[7]^

The study by Dällenbach et al which also found no difference between oral misoprostol and vaginal dinoprostone.^[8]^ They used 20 mcq to 40 mcq administered orally every 2 hours with a maximal total dose of 475 mcg versus 2 mg dinoprostone gel administered twice 6 hours apart for the other group.

While it appears that more doses are needed when using oral misoprostol than vaginal dinoprostone, the need for additional oxytocin is more in vaginal dinoprostone groups making this benefit also doubtful.

Vaginal misoprostol has been questioned for safety, especially for the fetus, the study by Chen et al^[63]^ reported an increased risk of chorioamnionitis, Garg et al explained that this is due to the repetitive vaginal examinations and doses required for vaginal misoprostol than those required for vaginal dinoprostone.^[10]^ While vaginal dinoprostone is still an available alternative for labor induction, it carries a high failure rate. Also, being more expensive than vaginal misoprostol limits its spread in some countries like India.^[11]^ Samanta et al mentioned that studies differed in their findings regarding the safety of vaginal misoprostol. Our meta-analysis confirms that vaginal misoprostol is safer for the fetus and less to lead to complications.

The study by Vimarshitha et al concluded that the use of a trans-cervical Foley catheter inflated with 30 to 50 mL normal saline taken out after 12 hours showed better results than the use of Intravaginal misoprostol or dinoprostone.^[52]^ The study by Young et al also concluded that no significant difference was found between oral misoprostol 50 mcq 4 hourly and low-dose vaginal misoprostol 25 to 50 mcq 2 doses 5 hours apart^[48]^

## 6. Limitations of the study

Our meta-analysis did not study the effects of different doses of vaginal misoprostol, however, a study by Hofmeyr et al found that outcomes didn’t differ too much except for the frequency of the need for additional oxytocin.^[64]^

## 7. Conclusion

Vaginal misoprostol is more effective at inducing labor but may be less safe than vaginal dinoprostone. Oral misoprostol is generally as safe as vaginal dinoprostone. Vaginal dinoprostone requires lower doses but may need more oxytocin administration.

## Author contributions

**Conceptualization:** Mohamed Ramadan.

**Data curation:** George Bashour.

**Project administration:** Fatma Labieb.

**Writing – original draft:** Mohamed Ramadan, George Bashour, Engy Eldokmery, Amnah Alkhawajah, Karim Alsalhi, Yara Badr, Asmaa Emad.

**Writing – review & editing:** Mohamed Ramadan, George Bashour, Engy Eldokmery, Amnah Alkhawajah, Fatma Labieb.
